# Detection of positive selection on depression-associated genes

**DOI:** 10.1038/s41437-025-00753-1

**Published:** 2025-03-13

**Authors:** Shiyu Yang, Chenqing Zheng, Canwei Xia, Jihui Kang, Langyu Gu

**Affiliations:** 1 Sun Yat-sen University Cancer Center, Guangzhou, Guangdong 510060 China; 2 The Affiliated Brain Hospital, Guangzhou Medical University, Guangzhou, Guangdong 510180 China; 3 State Key Laboratory for Biocontrol, School of Life Sciences, Sun Yat-sen University, Guangzhou, Guangdong 510275 China; 4 Ministry of Education Key Laboratory for Biodiversity and Ecological Engineering, College of Life Sciences, Beijing Normal University, Beijing, 100875 China; 5 Department of Pathology, The First Affiliated Hospital, Sun Yat-sen University, Guangzhou, Guangdong 510080 China

**Keywords:** Molecular evolution, Evolutionary biology

## Abstract

Although depression significantly impacts fitness, some hypotheses suggest that it may offer a survival benefit. However, there has been limited systematic investigation into the selection pressures acting on genes associated with depression at the genomic level. Here, we conducted comparative genomic analyses and computational molecular evolutionary analyses on 320 depression-associated genes at two levels, i.e., across the primate phylogeny (long timescale selection) and in modern human populations (recent selection). We identified seven genes under positive selection in the human lineage, and 46 genes under positive selection in modern human populations. Most positively selected variants in modern human populations were at UTR regions and non-coding exons, indicating the importance of gene expression regulation in the evolution of depression-associated genes. Positively selected genes are not only related to immune responses, but also function in reproduction and dietary adaptation. Notably, the proportion of depression-associated genes under positive selection was significantly higher than the positively selected genes at the genome-wide average level in African, East Asian, and South Asian populations. We also identified two positively selected loci that happened to be associated with depression in the South Asian population. Our study revealed that depression-associated genes are subject to varying selection pressures across different populations. We suggest that, in precision medicine—particularly in gene therapy—it is crucial to consider the specific functions of genes within distinct populations.

## Introduction

Depression ranks as the leading cause of disability and seriously affects the normal life of many people (Beurel et al. 2020). According to its typical clinical features, depression has multiple negative effects on human beings. The International Classification of Diseases (ICD-10-coded) defined depression as a mental disease in which *the patient suffers from lowering of mood, reduction of energy, and decrease in activity. (Capacity for enjoyment, interest, and concentration is reduced, and marked tiredness after even minimum effort is common. Sleep is usually disturbed and appetite diminished. Self-esteem and self-confidence are almost always reduced and, even in the mild form, some ideas of guilt or worthlessness are often present. The lowered mood varies little from day to day, is unresponsive to circumstances and may be accompanied by so-called “somatic” symptoms)* (https://icd.who.int/browse10/2016/en#/F32). Recent studies have also reported that most suicides have been diagnosed with depression (Hawton et al. 2013). In general, depression has considerable negative consequences, including notable costs to survival and fitness.

Although depression considerably impairs fitness, a few hypotheses propose that depression can have survival benefits. Some hypotheses focus on the phenotype of depression itself, emphasizing that emotions can affect physiological reactions and behavior, playing adaptive roles in social benefits, such as to reduce costly conflict and to avoid infection (Anders et al. 2013). Other hypotheses emphasize the associations between depression and immune functions, such as the pathogen‒host defense hypothesis (Raison and Miller 2013), which proposes that depression can help energy conservation and reallocation to enhance immune responses. To better understand the evolution of depression, we first need to systematically investigate the selection pressures on depression-associated genes at the genomic level.

Natural selection is the major force driving the adaptive evolution of human beings, and selection pressures can occur at different stages during evolution. On the one hand, selection at the species level must be considered on a long timescale, i.e., millions of years and usually working on the decisive phenotypes for speciation. For example, brain development and walking upright have been reported to be under positive selection in the human lineage (Wang and Crompton 2004; Dumas et al. 2021). On the other hand, migration out of Africa in the last 100,000 years has further shaped different modern human populations under different selection pressures, including environmental changes, diversified food resources, and new pathogens (Benton et al. 2021). These recent selection pressures work on new genotypes or gene networks that are adaptable. A series of studies have reported that genes involved in local adaptation, such as anti-UV radiation (Yang et al. 2022), pathogen antagonism (Klunk et al. 2022), and dietary adaptation (Bersaglieri et al. 2004; Kothapalli et al. 2016; Ye et al. 2017; Chen et al. 2022) are under positive selection. If these genes are also involved in diseases due to genetic pleiotropic effects, then disease-associated genes could also be under positive selection. Whether this is the case in depression-associated genes remains unknown.

Growing evidence has shown that depression has a polygenic genetic basis. With the rapid development of next generation sequencing technology, researchers have started to screen depression-associated genes at the whole-genomic level using genome-wide association studies (GWAS). Hundreds of genes have been identified to be associated with depression by using large cohorts from the UK Biobank, the Million Veteran Program, 23andMe, and FinnGen (Howard et al. 2018a, 2019; Wingo et al. 2021; Levey et al. 2021). Well-assembled and annotated genomes of multiple primate species are also available. These provide good resources for us to examine the selection pressures on the evolution of depression-associated genes in depth. Therefore, in this study, we investigated the positive selection on depression-associated genes retrieved from the GWAS studies mentioned above at two different levels, i.e., across the primate phylogeny (long timescale selection, i.e., millions of years) and in modern human populations (recent selection, about 100,000 years).

## Materials and methods

### Positive selection detection across the phylogeny

**Fig. 1:**
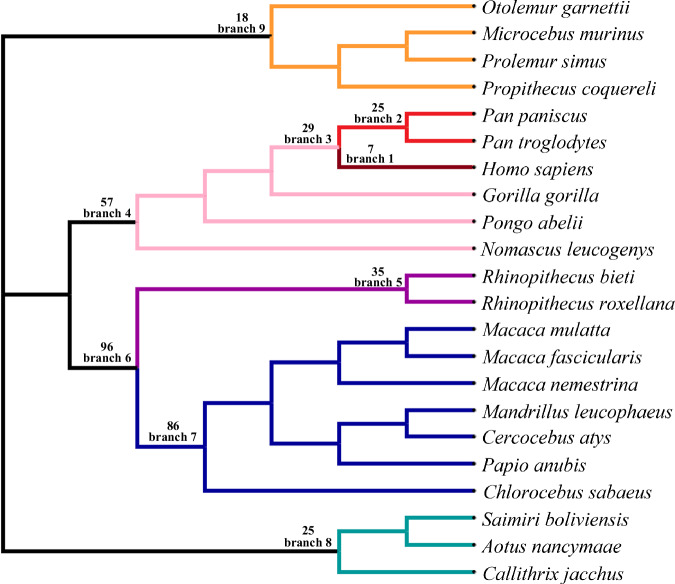
Positive selection detection of depression-associated genes across the primate phylogeny under the branch-site model implemented in PAML. Nine lineages were tested. The number of depression-associated genes under positive selection were given on each lineage.

320 depression-associated genes at autosomes identified by GWAS were retrieved from literatures (Howard et al. 2018a, 2019; Wingo et al. 2021; Levey et al. 2021) (Supplementary File 1). Ensembl IDs of human genes and corresponding orthologous genes of 21 primates across genomes were retrieved from the Ensembl database using BioMart (Cunningham et al. 2022). Only one-to-one orthologous genes with high confidence were analyzed. Coding genes and gff files for each species retrieved from the Ensembl database were used to extract corresponding coding sequences. The longest transcript of each gene was used for further analyses. Codon alignments were conducted using MAFFT implemented in T-coffee (Notredame et al. 2000) with default parameters. An unrooted tree retrieved from the literature (Upham et al. 2019) was used for PAML analyses (Fig. 1).

The branch-site model in PAML was used to test positive selection across the phylogeny, which consider signals among both lineages and sites (Yang 1997, 2007). The value of *ω* represents the ratio of nonsynonymous to synonymous substitutions. If the *ω* >>1, it means that the nonsynonymous mutations were favored by seletion. We detect positive selection on nine primate lineages (Fig. 1). To run the branch-site model in PAML, all branches within the target lineage were labeled as foreground branches. The M0 model was used to estimate initial branch lengths (fix_blength = 2). Alignment gaps and ambiguity characters were removed (Cleandata = 1). The modified Model A (model = 2, NSsites = 2) and the Model B (fix_omega = 1, omega = 1) were compared and a likelihood ratio test (LRT) was used to detect significance (Yang 1997, 2007). Multiple test corrections were conducted using Bonferroni correction (Anisimova and Yang 2007; Gu and Xia 2019; Gu et al. 2023).

### Positive selection detection in modern human populations

Haplotype-based methods (the integrated haplotype score (iHS) and the cross-population extended haplotype homozygosity (xpEHH)) which can limit demographic effects (Sabeti et al. 2002; Voight et al. 2006; Nielsen et al. 2007), as well as pairwise *F*_*ST*_ tests (Innan and Kim 2008) were used to detect positive selection across the genome in different modern human populations, retrieved from the 1000 Genomes Project (ftp.1000genomes.ebi.ac.uk/vol1/ftp/release/20130502/). The iHS test can detect positively selected genes that have not been fixed in the population, and the xpEHH test can detect positively selected genes that are near/at fixation (Voight et al. 2006; Sabeti et al. 2007). To further limit demographic effects, we focused on macro-populations, i.e. the African population, the East Asian population, the European population, and the South Asian population. We also removed recently admixed populations and geographically adjacent populations. Populations that we used were the same as the literature (Gu et al. 2023).

The vcf files of autosomes retrieved from the 1000 Genomes Project were processed with PLINK (Purcell et al. 2007; Chang et al. 2015) and VCFtools (Danecek et al. 2011). The GRCh37/hg19 genome was used as the reference. SNPs with indels were not used. Since allele frequency and *F*_*ST*_ are highly correlated, we grouped *F*_*ST*_ values in different allele frequency bins to define the *F*_*ST*_ threshold in each bin, and the top 5% *F*_*ST*_ was used as the threshold. *F*_*ST*_ and allele frequencies were calculated with VCFtools (Danecek et al. 2011). The selscan software was used to calculate and standardize iHS and xpEHH scores for each SNP across the genome with default parameters (Szpiech and Hernandez 2014). Only biallelic SNPs with minor allele frequency ≥ 0.05 in test populations were considered. The number of SNPs with ∣iHS∣or ∣xpEHH∣ > 2 was counted in a 51-SNP window. When under positive selection, SNPs with large absolute scores of iHS or xpEHH tend to be clustered together (Voight et al. 2006). Only SNPs with ∣iHS∣or ∣xpEHH∣ > 2 in a top 1% 51-SNP window, as well as with high *F*_*ST*_ values were considered under positive selection. Given the use of genome-wide empirical distributions for standardization, no formal significance testing was conducted when employing these haplotype-based methods, as noted in the literature (Voight et al. 2006). We focused on SNPs located in exons in this study. After identifying positively selected genes across the genome in different populations, we compared this gene list with depression-associated genes we have retrieved to identify which depression-associated genes were under positive selection. GO annotations of positively selected genes were conducted with DAVID (Huang et al. 2009; Sherman et al. 2022).

### Statistical analyses at the genomic level

Fisher tests were conducted for pairwise comparisons between humans and other primates to determine whether the proportion of positively selected depression-associated genes was significantly different. To determine whether the proportion of positively selected depression-associated genes was significantly larger than the proportion of positively selected genes at the genomic level (at the autosomal level) in the target branch, we randomly selected 320 genes (the same number as depression-associated genes we tested) across the genome using homologous genes retrieved for PAML analyses each time, and recorded how many of them were under positive selection in that branch. We repeated these steps 100,000 times for each branch and plotted the density distribution. Then, we checked whether the actual number of positively selected depression-associated genes was extremely high (located outside the 5% tails of the distribution).

To determine whether the proportion of positively selected depression-associated genes in the target population was significantly larger than the proportion of positively selected genes at the genomic level (at the autosomal level) in that population, we randomly selected 320 genes (the same number as depression-associated genes we tested) across the genome each time and recorded how many of them were under positive selection in that population. We repeated these steps 100,000 times for each population and plotted the density distribution. Then we checked whether the actual number of positively selected depression-associated genes was extremely high (located outside the 5% tails of the distribution).

### Gene Ontology annotations

Gene Ontology biological process terms were retrieved from AmiGO (https://amigo.geneontology.org/amigo/) (Ashburner et al. 2000; Carbon et al. 2009; Aleksander et al. 2023).

### Expression quantitative trait loci (eQTLs) for positively selected variants

To see whether positively selected SNPs function as eQTLs, the GTExPortal V8 (https://www.gtexportal.org/home/) was used to acquire corresponding information (Ardlie et al. 2015). The m-value (Posterior probability from MetaSoft) was used to measure the eQTL effects in multiple tissues (Han and Eskin 2011, 2012). If m > 0.9, it means that the variant does have an eQTL effect in the tissue.

## Results

### Depression-associated genes under positive selection across the primate phylogeny

Among the 320 depression-associated genes we tested, seven genes were under positive selection in the human lineage (Table 1, Fig. 1), including the immune response-related gene STAU1 (Pang et al. 2021), the neurodegeneration-related gene PSEN2 (Fedeli et al. 2019), the neurological-related gene ANKK1 (Hoenicka et al. 2010), the electron transfer flavoprotein dehydrogenase ETFDH (Zhang and Zhao 2022), zinc fingers and homeoboxes 3 ZHX3, the neural development-related gene PCDH9 (Chen et al. 2024), and immune-related gene LYRM4 (Wang et al. 2023).

**Table 1:** Depression-associated genes under positive selection in the human lineage.

	Branch-site model				Sites under positive selection
Gene	Model A	Model B	w	*p*/2	Under BEB model with a posterior probability > 0.95
STAU1	−4133.6	−4152.73	103.19	0	96E
PSEN2	−4314.65	−4345.9	62.51	0	392S, 393C, 397W, 398S, 400M, 402R, 407W, 408P, 411F, 413C
ANKK1	−6287.36	−6302.23	29.74	2.47E-08	N/A
ETFDH	−4791.55	−4847.84	112.59	0	88V,428N,429L,430S,433N,434E,436M,437T,438V,440K,442G,446S,447V,448S
					450T,451L,452D,453S,454E,455T,456T,457Q,458L,460T,465F,466I,467M,468Y
ZHX3	−7572.95	−7586.06	26.22	1.53E-07	N/A
PCDH9	−6267.04	−6275.47	16.85	0.00002	N/A
LYRM4	−381.331	−387.241	11.82	0.00029	11S

Tests were conducted under the branch-site model implemented in PAML.

Compared to the human lineage, more depression-associated genes were identified under positive selection in other lineages. For example, 25 depression-associated genes were under positive selection in branch 2, which is the sister lineage to human beings, including *Pan troglodytes* and *Pan paniscus*. Twenty-nine depression-associated genes were under positive selection in branch 3, the ancestral lineage leading to human beings. Moreover, there were 57 depression-associated genes under positive selection in branch 4, 35 depression-associated genes under positive selection in branch 5, 96 depression-associated genes under positive selection in branch 6, 86 depression-associated genes under positive selection in branch 7, 25 depression-associated genes under positive selection in branch 8, and 18 depression-associated genes under positive selection in branch 9 (Fig. 1).

**Fig. 2:**
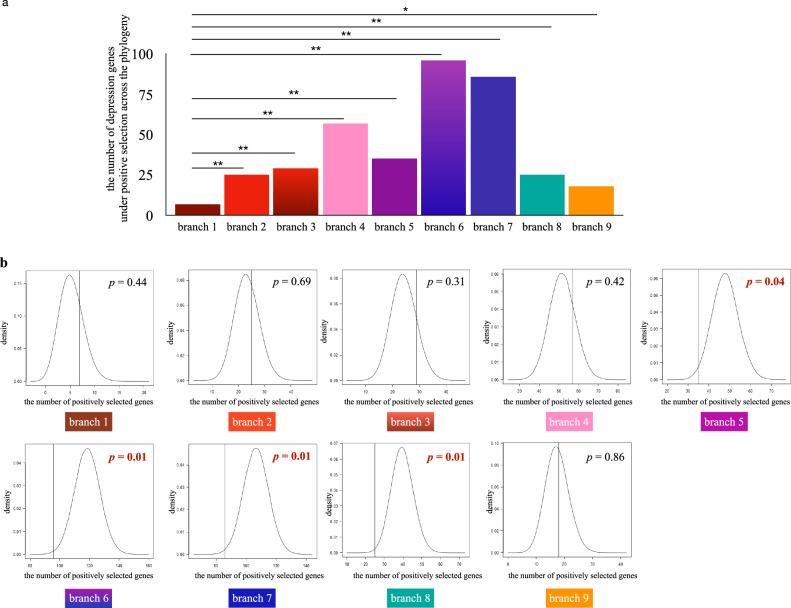
Statistical analyses of depression-associated genes under positive selection in different primate lineages. **a** Statistical analyses showed that the number of positively selected depression-associated genes in non-human primate lineages were significantly larger than the one in the human lineage. **b** The proportion of positively selected depression-associated genes was not significantly larger than the proportion of positively selected genes at the genomic level in target primate lineages. * represents *p* < 0.05; ** represents *p* < 0.01.

We further tested whether the proportion of positively selected depression-associated genes was significantly larger than the proportion of positively selected genes at the whole genomic level. We performed statistical significance tests in all target lineages and did not find any significantly large result. In most cases, there were no significant differences, and the proportion of positively selected depression-associated genes was even significantly less than the proportion of positively selected genes at the genomic level in certain lineages (branch 5, branch 6, branch 7 and branch 8) (Fig. 2).

### Depression-associated genes under positive selection in modern human populations

Genome-wide positive selection detection identified 215 genes under positive selection in the African population, 592 genes under positive selection in the East Asian population, 632 genes under positive selection in the European population, and 567 genes under positive selection in the South Asian population. We further identified 46 depression-associated genes under positive selection in total. Among them, nine depression-associated genes were identified under positive selection by both the iHS test and the *F*_*ST*_ test, and 45 depression-associated genes were identified under positive selection by both the xpEHH test and the *F*_*ST*_ test (Table 2, Supplementary File 2). Depression-associated genes under positive selection exhibited population-specific patterns. There were eight genes (HLA-DQA1, HLA-DQB1, MGAT4C, ZNF536, SIM1, METTL9, MYRF, PSORS1C1) under positive selection in the African population, five genes (BEND4, CDH13, SOX6, POGZ, PSMB4) under positive selection in the European population, 17 genes (SDK1, CCDC92, DENND1A, USP3, C22orf26, BEND4, ASXL3, TENM2, BSN, ZNF35, MGAT4C, CTNND1, FNIP2, NRD1, INPP4B, C7orf72, FHIT) under positive selection in the East Asian population, and 25 genes (FADS1, FADS2, HLA-DQA1, HLA-DQB1, FAM120A, DENND1A, C22orf26, BEND4, FAM120AOS, ASCC3, LPIN3, POGZ, EMILIN3, CDH9, SGIP1, ZNF660, NICN1, CCDC36, CCDC71, TCAIM, MGAT4C, ZNF445, ZNF197, ZKSCAN7, PHF2) under positive selection in the South Asian population (Table 2, Supplementary File 2). FADS1 and FADS2 which are related to dietary adaptation have been previously reported to be under positive selection (Kothapalli et al. 2016; Ye et al. 2017; Chen et al. 2022).

**Table 2:** Depression-associated genes under positive selection in different human populations.

Ensembl ID	gene	GO_BP term	
ENSG00000196735	HLA-DQA1	adaptive immune response GO:0002250	Africa
ENSG00000179344	HLA-DQB1	adaptive immune response GO:0002250	
ENSG00000182050	MGAT4C	viral protein processing GO:0019082	
ENSG00000198597	ZNF536	negative regulation of neuron differentiation GO: 0045665	
ENSG00000112246	SIM1	nervous system development GO:0007399	
ENSG00000197006	METTL9	protein methylation GO:0006479	
ENSG00000124920	MYRF	central nervous system myelination GO:0022010	
ENSG00000204540	PSORS1C1	N/A	
ENSG00000188848	BEND4	N/A	Europe
ENSG00000140945	CDH13	mitotic cell cycle GO:0000278	
ENSG00000110693	SOX6	central nervous system development GO:0007417	
ENSG00000143442	POGZ	DNA repair GO:0006281	
ENSG00000159377	PSMB4	negative regulation of inflammatory response to antigenic stimulus GO:0002862	
ENSG00000146555	SDK1	synapse assembly GO: 0007416	East Asia
ENSG00000119242	CCDC92	innate immune response GO: 0045087	
ENSG00000119522	DENND1A	endocytosis GO:0006897	
ENSG00000140455	USP3	DNA repair GO: 0006281	
ENSG00000182257	C22orf26/PRR34	N/A	
ENSG00000188848	BEND4	N/A	
ENSG00000141431	ASXL3	DNA-templated transcription GO: 0006351	
ENSG00000145934	TENM2	neuron development GO: 0048666	
ENSG00000164061	BSN	chemical synaptic transmission GO:0007268	
ENSG00000169981	ZNF35	regulation of DNA-templated transcription GO: 0006355	
ENSG00000182050	MGAT4C	viral protein processing GO:0019082	
ENSG00000198561	CTNND1	brain development GO: 0007420	
ENSG00000052795	FNIP2	negative regulation of transcription by RNA polymerase II GO: 0000122	
ENSG00000078618	NRD1/NRDC	positive regulation of axonogenesis GO: 0050772	
ENSG00000109452	INPP4B	phosphatidylinositol biosynthetic process GO: 0006661	
ENSG00000164500	C7orf72/SPATA48	spermatogenesis GO: 0007283	
ENSG00000189283	FHIT	purine nucleotide metabolic process GO: 0006163	
ENSG00000149485	FADS1	lipid metabolic process GO: 0006629	South Asia
ENSG00000134824	FADS2	lipid metabolic process GO: 0006629	
ENSG00000196735	HLA-DQA1	adaptive immune response GO:0002250	
ENSG00000179344	HLA-DQB1	adaptive immune response GO:0002250	
ENSG00000048828	FAM120A	N/A	
ENSG00000119522	DENND1A	endocytosis GO:0006897	
ENSG00000182257	C22orf26/PRR34	N/A	
ENSG00000188848	BEND4	N/A	
ENSG00000188938	FAM120AOS	N/A	
ENSG00000112249	ASCC3	DNA dealkylation involved in DNA repair GO: 0006307	
ENSG00000132793	LPIN3	fatty acid catabolic process GO: 0009062	
ENSG00000143442	POGZ	DNA repair GO:0006281	
ENSG00000183798	EMILIN3	N/A	
ENSG00000113100	CDH9	synapse assembly GO:0007416	
ENSG00000118473	SGIP1	response to dietary excess GO:0002021	
ENSG00000144792	ZNF660	regulation of transcription by RNA polymerase II GO:0006357	
ENSG00000145029	NICN1	N/A	
ENSG00000173421	CCDC36/IHO1	synapsis GO: 0007129	
ENSG00000177352	CCDC71	N/A	
ENSG00000179152	TCAIM	N/A	
ENSG00000182050	MGAT4C	viral protein processing GO:0019082	
ENSG00000185219	ZNF445	regulation of DNA-templated transcription GO: 0006355	
ENSG00000186448	ZNF197	regulation of DNA-templated transcription GO: 0006355	
ENSG00000196345	ZKSCAN7	regulation of DNA-templated transcription GO: 0006355	
ENSG00000197724	PHF2	DNA-templated transcription GO: 0006351	

**Fig. 3:**
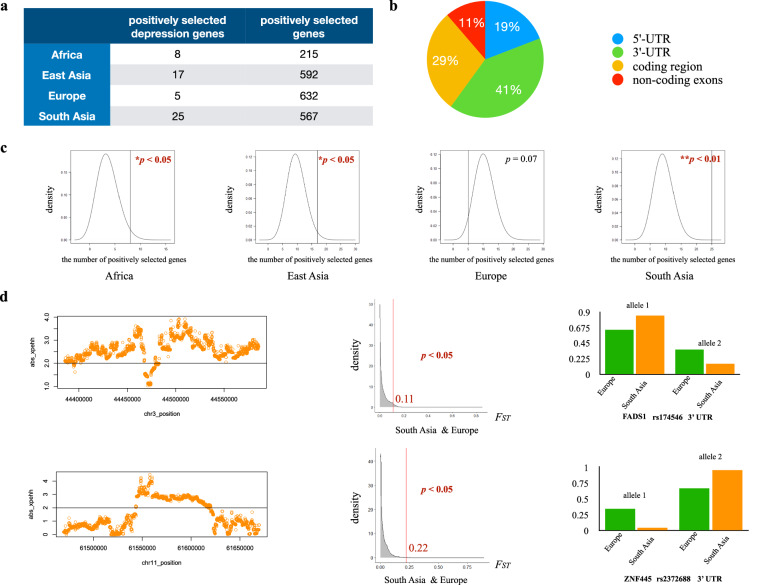
Recent selection plays important roles on the adaptive evolution of depression-associated genes in modern human populations. **a** The number of positively selected depression-associated genes and the number of positively selected genes at the genomic level in each population. **b** Proportions of positively selected variants located in different regions (5’ UTR, 3’ UTR, coding region and non-coding exons). **c** The proportion of positively selected depression-associated genes in the African population, the East Asian population and the South Asian population was significantly larger than the proportion of positively selected genes at the corresponding genomic level. **d** left: xpEHH plot of two positively selected variants that happened to be depression-associated variants in the South Asian population compared to the European population. middle: the distribution of *F*_*ST*_ values across the genome between the South Asian population and the European population. The *F*_*ST*_ value of two positively selected variants was located at the top 5% region of the corresponding distribution (*p* < 0.05). right: allele frequencies of two positively selected variants in the European population and in the South Asian population.

Notably, the proportions of depression-associated genes under positive selection in the African population (8 out of 320) (*p* < 0.05), in the East Asian population (17 out of 320) (*p* < 0.05), and in the South Asian population (25 out of 320) (*p* < 0.01) were significantly larger than the proportions of positively selected genes at the genomic level in the corresponding populations (Fig. 3).

### Expression quantitative trait loci (eQTLs) for positively selected variants

**Fig. 4:**
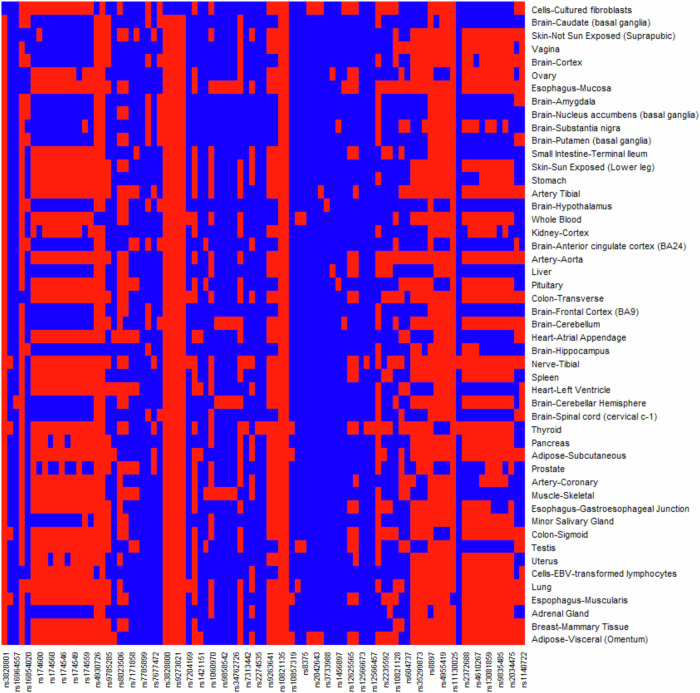
The eQTL effects of each positively selected variant in multiple tissues. The m-value was used to measure the eQTL effects in multiple tissues (Han and Eskin 2011, 2012). If m > 0.9, it means that the variant does have an eQTL effect in the tissue. red, m > 0.9; blue, m ≤ 0.9. Only variants with available information in the GTEx database were shown.

The eQTL effects of each positively selected variant at UTR regions were obtained from the GTExPortal V8. Results showed that most positively selected variants have eQTL effects across multiple tissues, and many of which were in non-brain tissues (Fig. 4).

## Discussion

We identified seven depression-associated genes under positive selection in the human lineage. Non-human primates showed more depression-associated genes under positive selection than humans. This phenomenon is consistent with findings indicating that humans have fewer genes under positive selection compared to non-human primates (Gibbs et al. 2007; Bakewell et al. 2007). There are several possible explanations for our findings. Recent studies indicate that brain evolution occurred not only in humans but also in non-human primates, such as the Simiiformes ancestor, which displayed significant morphological changes, including rapid brain expansion (Hu et al. 2024). Primate brain evolution has been a continuous process marked by the expansion of the neocortex and the development of higher cognitive functions (Zhuang et al. 2023; Hu et al. 2024). Another explanation could be gene pleiotropy. Many positively selected genes in non-human primates are involved in functions beyond brain development, such as reproduction, metabolism, dietary adaptation, and immunity (Supplementary 3). Similar patterns have been observed in cancer-associated (Lou et al. 2014; Vicens and Posada 2018; Gu et al. 2023) and Alzheimer’s disease-associated genes (Vamathevan et al. 2008). Additionally, environmental and societal pressures differ between humans and non-human primates, leading to distinct selective pressures on the same gene functions. For example, genes related to emotional regulation may promote social bonding in non-human primates (Platt et al. 2016), whereas excessive emotional sensitivity may lead to stress and negative effects which could be detrimental in humans (Dosari et al. 2023), making such traits less advantageous.

The seven positively selected depression-associated genes we identified in the human lineage are involved in brain development (PSEN2, ANKK1, PCDH9) and immunity (STAU1 and LYRM4), as highlighted in our results. It has been demonstrated that PSEN2 knock-in mice exhibit severe depressive behavior (Yoo et al. 2024), and PCDH9 and ANKK1 have also been reported as risk genes for depression (Roetker et al. 2012; Xiao et al. 2018). The positive selection of these genes in the human lineage may attributed to their pleiotropic effects. For example, brain-related genes have long been associated with cognitive evolution in humans (Zhuang et al. 2023; Hu et al. 2024), while immunity likely played a role in responding to new environments and pathogens during human evolution (Benton et al. 2021).

Notably, among the 46 depression-associated genes under positive selection in modern human populations, we identified two genes harboring depression-associated SNPs that are also under positive selection (Fig. 3). One is located in the gene FADS1. The odds ratio (OR) for allele T on rs174546 of FADS1 was significantly greater than 1 (OR = 1.015, *p* = 0.000856) (Howard et al. 2018b), indicating an increased risk of depression. Despite this, the locus is under positive selection, suggesting it may provide an adaptive advantage in certain environmental or evolutionary contexts. FADS1 is known for its role in dietary adaptation in South Asian population (Kothapalli et al. 2016; Ye et al. 2017; Chen et al. 2022), supporting the biosynthesis of long-chain polyunsaturated fatty acids, which are crucial for individuals on plant-based diets. This gene’s pleiotropic trade-off could explain the positive selection of this depression-associated locus in South Asians. Another positively selected depression-associated SNP is located in the gene ZNF445, which is involved in post-fertilization methylation maintenance and imprinting disorders (Takahashi et al. 2019). Unlike the SNP in FADS1, the SNP in ZNF445 has an OR for allele T on rs2372688 of 0.98 (*p* = 0.000006758) (Howard et al. 2018b), suggesting a protective effect against depression. Carriers of this variant may be less prone to depression and better adapted to environmental or social conditions, enhancing their survival and reproductive success. Further research is needed to explore the detailed function of this variant. Together, these two variants offer valuable complementary evidence that disease (depression)-associated loci can undergo positive selection in specific populations.

For the additional 44 depression-associated genes detected under positive selection in different human populations, although the loci subjected to positive selection are not directly linked to depression but rather to other loci within the genes, the positive selection of these genes may have led to their wider prevalence across human populations over time. This increased frequency could provide more opportunities for the associated depression-related variants to exert their effects, whether by indirectly promoting or inhibiting depression. Most of these genes are not only involved in neuron development, but also play roles in immune response, metabolism, dietary excess, and reproduction (e.g., spermatogenesis) (see Table 2). As we know, the environment has changed a lot after migration out of Africa in the last 100,000 years, such as exposure to diverse pathogens, diversified food resources, and social culture (Benton et al. 2021). In fact, many of the depression-associated genes that underwent positive selection in both African and South Asian populations are immune-related, such as HLA-DQA1, HLA-DQB1, and MGAT4C. Given that Africa and South Asia are located in tropical regions with greater diversity of pathogens and parasites compared to other populations, these genes could be under positive selection due to their association with immune functions. Additionally, some depression-associated genes in the South Asian population are linked to diet and metabolism, such as FADS1, FADS2, LPIN3, and SGIP1. As we mentioned above, FADS1 and FADS2 are well-known for their roles in dietary adaptation in the South Asian population (Kothapalli et al. 2016; Ye et al. 2017; Chen et al. 2022). These depression-associated genes may therefore under positive selection in modern human populations due to their pleiotropic effects.

Our results also showed that most positively selected variants were located in UTR regions and non-coding exons. In recent years, there has been increasing recognition that not all exons encode proteins (Aspden et al. 2023). For instance, a study has demonstrated that multiple non-coding exons within the brain-derived neurotrophic factor (BDNF) can produce differently spliced transcripts, potentially influencing psychiatric diseases like depression (Begni et al. 2017). Currently, research on non-coding exons in relation to depression is limited. Therefore, the positively selected sites in non-coding exons we have identified here are good candidates for further study. Regarding the UTR regions, the 5’-UTR harbors various regulatory elements, including sites for ribosome recruitment, binding sites for microRNAs, and structural components crucial for governing mRNA stability, pre-mRNA splicing, and the initiation of translation (Ryczek et al. 2023). Consequently, the 5’-UTR plays a crucial role in gene expression control. While their link to depression remains relatively sparse, one study has proposed that epigenetic methylation in the 5’-UTR region could influence glucocorticoid receptor splicing, which is implicated in depression (Turner et al. 2010). For 3’-UTR regions, they are renowned for providing binding sites for small RNAs, thus regulating gene expression at the post-transcriptional level, which significantly contributes to controlling behaviors like social interactions and anxiety, relevant to depression (Narayanan and Schratt 2020). This has been underscored by various studies, such as alternative splicing of the serotonin-1A receptor (Le François et al. 2018) and the regulation of VGF protein expression, a precursor of secreted neuropeptides (Lin et al. 2021). Therefore, the positively selected SNPs identified in UTR regions and non-coding exons in our study represent promising candidates for understanding their potential roles in depression. Besides, most of the positively selected depression-associated genes had eQTL effects in multiple tissues, especially in non-brain tissues, confirming again their pleiotropic effects.

We further revealed that the proportion of positively selected depression-associated genes was so high that it was even significantly larger than the proportions of positively selected genes at the genomic level in several modern human populations, i.e., in the African population, the East Asian population and the South Asian population, but not in the European population. As shown in Table 2, many positively selected genes in non-European populations are linked to immunity, neurodevelopment, metabolism, dietary adaptation, and reproduction. The disparities between non-European and European populations may stem from several factors. For example, diets in Africa, South Asia, and East Asia are more plant-based than in Europe. Social structures in these regions are also more conservative and community-oriented. And non-European populations face greater social competition due to economic growth, population density, and higher biodiversity. However, as mentioned above, most of the positively selected loci are not directly associated with depression, but rather are other loci within depression-associated genes. Therefore, we cannot conclude whether depression is adaptive in different populations at this stage. What we can say here is that these depression-associated genes can experience different selective pressures across populations, and these pressures are closely linked to their pleiotropic effects.

It is important to note that the depression-associated genes we tested were retrieved from individuals of European descent (Howard et al. 2018a, 2019; Wingo et al. 2021; Levey et al. 2021). However, GWAS data from non-European populations are currently limited, which restricts our ability to conduct a more comprehensive analysis. Future research, when relevant data becomes available, could provide further insights. Despite these limitations, our current study provides a valuable resource and logical framework for future analyses. Moreover, our research highlights that depression-associated genes, or more broadly, disease-associated genes, can be subject to different selective pressures in different populations, and even undergo positive selection. For example, one of the risk locus in FADS1 we identified, significantly associated with depression, was under positive selection in a specific population. This suggests that in precision medicine research, it is important to take into account the specific factor of how genes exert their functions in particular populations.

## Supplementary information

Supplementary File 1

Supplementary File 2

Supplementary File 3

## Data Availability

All data generated or analyzed during this study are included in this published article and its supplementary information files. Phased genetic data of the 1000 Genomes Project of modern human populations were retrieved from ftp.1000genomes.ebi.ac.uk/vol1/ftp/release/20130502/.
